# *In Vivo* Biotinylation of the *Toxoplasma* Parasitophorous Vacuole Reveals Novel Dense Granule Proteins Important for Parasite Growth and Pathogenesis

**DOI:** 10.1128/mBio.00808-16

**Published:** 2016-08-02

**Authors:** Santhosh M. Nadipuram, Elliot W. Kim, Ajay A. Vashisht, Andrew H. Lin, Hannah N. Bell, Isabelle Coppens, James A. Wohlschlegel, Peter J. Bradley

**Affiliations:** a Department of Microbiology, Immunology and Molecular Genetics, University of California, Los Angeles, Los Angeles, California, USA; b Department of Pediatrics, David Geffen School of Medicine at the University of California, Los Angeles, Los Angeles, California, USA; c Department of Biological Chemistry and Institute of Genomics and Proteomics, University of California, Los Angeles, Los Angeles, California, USA; d Department of Molecular Microbiology and Immunology, The Johns Hopkins University Bloomberg School of Public Health, Baltimore, Maryland, USA; e Molecular Biology Institute, University of California Los Angeles, Los Angeles, California, USA

## Abstract

*Toxoplasma gondii* is an obligate intracellular parasite that invades host cells and replicates within a unique parasitophorous vacuole. To maintain this intracellular niche, the parasite secretes an array of dense granule proteins (GRAs) into the nascent parasitophorous vacuole. These GRAs are believed to play key roles in vacuolar remodeling, nutrient uptake, and immune evasion while the parasite is replicating within the host cell. Despite the central role of GRAs in the *Toxoplasma* life cycle, only a subset of these proteins have been identified, and many of their roles have not been fully elucidated. In this report, we utilize the promiscuous biotin ligase BirA* to biotinylate GRA proteins secreted into the vacuole and then identify those proteins by affinity purification and mass spectrometry. Using GRA-BirA* fusion proteins as bait, we have identified a large number of known and candidate GRAs and verified localization of 13 novel GRA proteins by endogenous gene tagging. We proceeded to functionally characterize three related GRAs from this group (GRA38, GRA39, and GRA40) by gene knockout. While Δ*gra38* and Δ*gra40* parasites showed no altered phenotype, disruption of *GRA39* results in slow-growing parasites that contain striking lipid deposits in the parasitophorous vacuole, suggesting a role in lipid regulation that is important for parasite growth. In addition, parasites lacking *GRA39* showed dramatically reduced virulence and a lower tissue cyst burden *in vivo*. Together, the findings from this work reveal a partial vacuolar proteome of *T. gondii* and identify a novel GRA that plays a key role in parasite replication and pathogenesis.

## IMPORTANCE

Most intracellular pathogens reside inside a membrane-bound vacuole within their host cell that is extensively modified by the pathogen to optimize intracellular growth and avoid host defenses. In *Toxoplasma*, this vacuole is modified by a host of secretory GRA proteins, many of which remain unidentified. Here we demonstrate that *in vivo* biotinylation of proximal and interacting proteins using the promiscuous biotin ligase BirA* is a powerful approach to rapidly identify vacuolar GRA proteins. We further demonstrate that one factor identified by this approach, GRA39, plays an important role in the ability of the parasite to replicate within its host cell and cause disease.

## INTRODUCTION

*Toxoplasma gondii* is an intracellular parasite that is capable of infecting virtually all warm-blooded animals and nearly any mammalian cell type (1). Human infection is estimated at approximately 30% of the world’s population, although rates vary widely, depending on geographical location (2). Most humans have no manifestation of chronic disease (asymptomatic infection), although in the acute phase, many will develop flu-like symptoms, including fever, lymphadenitis, and fatigue. Immunocompromised individuals, such as those with HIV infection, solid organ or hematopoietic stem cell transplant, or those on high-dose steroid therapy, are subject to dire end organ diseases, including eye disease (retinitis) and central nervous system disease (encephalitis) (3). Fetuses of mothers with acute or reactivated toxoplasmosis are also at risk of congenital infection, with manifestations ranging from retinitis to devastating cerebritis and obstructive hydrocephalus with consequent global mental and physical disability (3). Despite our knowledge of this disease over the last 100 years, there is still much to learn about how *Toxoplasma* invades host cells, establishes a replication-competent niche, and acquires nutrients from its host cell.

*Toxoplasma* invasion is mediated by three specialized secretory organelles, named the micronemes, rhoptries, and dense granules, which contribute to the parasite’s ability to initiate and sustain infection within its host (4). The micronemes first secrete an array of adhesins that facilitate parasite attachment to the surface of the host cell (5). Second, the club-shaped rhoptries secrete proteins that enable host cell penetration and vacuole formation, as well as hijacking of host immune functions (6). Finally, proteins from the dense granules are secreted which are implicated in the remodeling and maintenance of the nascent parasitophorous vacuole (PV) for intracellular survival (7). Although the dense granule proteome is hypothesized to be composed of hundreds of proteins, only about 30 of these proteins have been discovered, and their precise roles in intracellular parasite survival and growth are not well elucidated (8). Thus far, it is known that some dense granule proteins (GRAs) are integral for the formation and maintenance of a lipid-based intravacuolar network (IVN), while others are responsible for the uptake of nutrients from the host cell (9–13). Recently, a newly discovered class of GRAs has been discovered that are exported beyond the vacuolar membrane into the host cytoplasm and modulate host immune and cell cycle activities (14–17).

To date, most GRAs have been discovered individually by subcellular fractionation of organelles and antibody production, analysis of excreted or secreted fractions, screening for immunogenic peptides, or bioinformatics searches for proteins containing secretory signal peptides (8, 18). Many of these previously identified GRAs are extremely abundant or immunogenic, which aided in their discovery. The more recently discovered class of host cell-exported GRAs were found *in silico* by screening for secreted proteins that also contain nuclear localization sequences suggestive of trafficking to the host cell nucleus or examining for secreted proteins with predicted interaction with the host immune machinery (14). Although these approaches have been successful, we sought a method to more rapidly identify a large number of PV components. This has been previously difficult as there is no effective method for purifying the PV without substantial parasite and/or host contamination.

To address this need, we applied the BioID technique to label dense granule proteins secreted into the PV. This biochemical approach utilizes a promiscuous biotin ligase that, when fused to a “bait” protein, can traffic to an organellar subcompartment and biotinylate multiple interacting and proximal proteins of the bait (19). Once biotinylated, the parasites are lysed, and the biotinylated proteins are purified using streptavidin affinity chromatography and identified by mass spectrometry. We have recently used this approach to identify a number of novel components of the *Toxoplasma* inner membrane complex (IMC) (20).

In this report, we show that the BioID system with dense granule bait proteins can successfully be used to biotinylate and identify a large number of known and candidate GRAs. From a subset of this candidate pool, we were able to thus far identify 13 novel GRAs (GRAs 28 to 40) using C-terminal endogenous gene tagging and immunofluorescence assays (IFAs), greatly expanding the known GRA proteome of *T. gondii*. We then conducted a functional analysis on a group of three related novel GRA proteins (GRAs 38, 39, and 40) and demonstrate that disruption of GRA39 results in the accumulation of lipid deposits in the parasitophorous vacuole and plays a key role in parasite replication, virulence, and cyst burden.

## RESULTS

### *In vivo* biotinylation of the PV using GRA proteins as bait.

**FIG 1:**
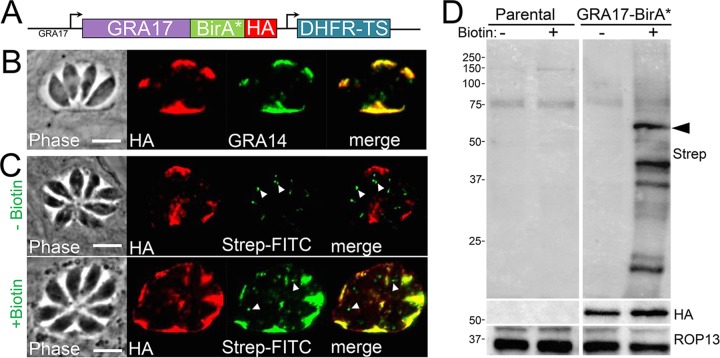
GRA17-BirA* localizes to the PV and can biotinylate proteins in the vacuole. (A) Diagram of the construct encoding the promoter and full genomic sequence of GRA17 fused to BirA* along with a 3×HA C-terminal epitope tag. (B) IFA showing that GRA17-BirA* traffics appropriately to the PV and colocalizes with GRA14. Scale bar, 10 µm (applicable to all panels in panel B). (C) IFA of GRA17-BirA* expressing parasites ± biotin, showing the PV is labeled in a biotin-dependent manner. Endogenously biotinylated apicoplasts are observed with and without biotin (arrowheads). Scale bar, 10 µm (applicable to all panels in panel C). (D) Western blot of whole-cell lysates of parental (RH Δ*hxgprt*) and GRA17-BirA*-expressing parasites ± biotin. Lysates were probed with streptavidin-HRP, revealing an increase in biotinylated proteins in GRA17-BirA*-expressing parasites upon addition of biotin. The GRA17-BirA* fusion protein is predicted to be ~59 kDa (arrowhead) (10).

To identify a large number of vacuolar resident proteins, we adapted the BioID approach using a secreted GRA protein as bait. To do this, we created a fusion of the vacuolar pore component GRA17 with the biotin ligase BirA* followed by a C-terminal three-hemagglutinin (3×HA) epitope tag (Fig. 1A) (10). When expressed in *Toxoplasma*, the GRA17-BirA* fusion trafficked appropriately to the PV and colocalized with the known dense granule protein GRA14 when examined by IFA, indicating that the fusion did not alter trafficking of GRA17 (Fig. 1B) (21). In addition, GRA17-BirA* labeled the PV only when biotin was supplemented (as assessed by streptavidin-fluorescein isothiocyanate [FITC] staining), demonstrating that the fusion is catalytically active in this compartment (Fig. 1C). By Western blotting, we found a dramatic increase in biotinylated proteins in GRA17-BirA* parasite lysates, confirming the ability of the GRA17-BirA* fusion to biotinylate a range of targets in the PV (Fig. 1D). Large-scale GRA17-BioID experiments were then performed on intracellular parasites with a high multiplicity of infection (~5), which were grown for ~36 h to obtain large vacuoles to maximize labeling of the PV. These intracellular parasites were harvested and lysed, and biotinylated proteins were purified via streptavidin chromatography.

Compared to a control lysate derived from wild-type parasites supplemented with biotin, the GRA17-BioID purified fraction revealed a large number of known dense granule proteins (e.g., GRAs, MAG1, MAF1, cathepsin, and nucleoside triphosphatase II [NTPase II]) (7, 8), all of which ranked highly by normalized spectral abundance factor (NSAF) (Table 1; see Table S1 in the supplemental material). We therefore concluded that GRA17-BirA* appropriately labeled PV-resident proteins. In total, the GRA17-BioID experiment yielded 279 proteins (see Table S1). While the highest-ranking proteins were mostly GRAs, the lower-ranked proteins also included other secreted proteins, such as rhoptry body proteins (ROPs) and rhoptry neck proteins (RONs), as well as some proteins from the micronemes (MICs) and parasite surface antigens (SAGs) that would be exposed to the PV.

**TABLE 1:** Summary of known and novel GRAs found by GRA17-BioID^a^

Gene ID (TgGT1 no. [ToxoDB version 7.3])	Gene ID (TgGT1 no. [ToxoDB release 25])	Protein name
065990	222170	GRA17
034740	288650	GRA12
083030	227620	GRA2
017550	203310	GRA7
082670	227280	GRA3
032340	290700	GRA25
108770	270240	MAG1
096230	297880	GRA23
**115760**	**232000**	**GRA30**
053770	220950	MAF1
068065	275440	GRA6
**014920**	**220240**	**GRA31**
021860	208830	GRA16
087090	310780	GRA4
**072150**	**212300**	**GRA32**
**025470**	**247440**	**GRA33**
064850	410370, 279100, 279100A, 279100B, 411470, 220950	MAF1-related protein
**017570**	**203290**	**GRA34**
000480	275860	GRA12 (paralog)
051100	239740	GRA14
053780	410370, 279100, 279100A, 279100B, 411470, 220950	MAF1-related protein
037870	286450	GRA5
004030	254470	MYR1
**081550**	**226380**	**GRA35**
053890	410360	MAF1-related protein
**039660**	**213067**	**GRA36**
108780	270250	GRA1
**115800**	**231960**	**GRA28**
004270	254720	GRA8
**069040**	**236890**	**GRA37**
**088710**	**312420**	**GRA38**
102940	251540	GRA9
**108120**	**269690**	**GRA29**
117710	230180	GRA24
065030	221210	Cyclophilin
068050	275470	GRA15
125960.0	215220	GRA22
**033840**	**289380**	**GRA39**

a Novel GRAs 28-40 are highlighted in boldface.

**FIG 2:**
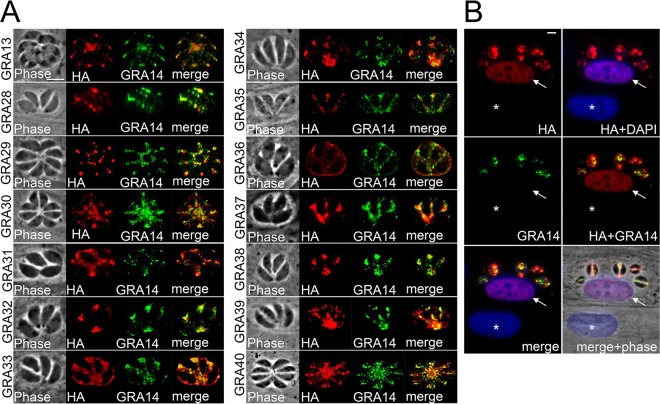
Identification of a novel dense granule protein, GRA13, and GRAs 30 to 40 by GRA17-BioID. (A) IFA with rabbit anti-HA antibodies shows strong staining of the parasitophorous vacuole for each novel GRA that colocalizes with GRA14, demonstrating that these are novel dense granule proteins. Gene numbers and novel designations are as follows: TgGT1_237880, GRA13; TgGT1_231960, GRA28; TgGT1_269690, GRA29; TgGT1_232000, GRA30; TgGT1_220240, GRA31; TgGT1_212300, GRA32; TgGT1_247440, GRA33; TgGT1_203290, GRA34; TGGT1_226380, GRA35; TgGT1_213067, GRA36; TgGT1_236890, GRA37; TgGT1_312420, GRA38; TgGT1_289380, GRA39; and TgGT1_219810, GRA40. Scale bar in upper left-hand panel (GRA13), 10 µm (applicable to all panels in panel A). (B) GRA28 is exported to the host cell and concentrates within the host nucleus. IFA with rabbit anti-HA antibody shows signal within the nucleus of a host fibroblast infected with GRA28-HA parasites (arrow). A neighboring uninfected host cell is shown for contrast (asterisk). Scale bar in upper left hand panel, 10 µm (applicable to all panels in panel B).

To expand our studies beyond labeling with GRA17, we generated two additional BioID constructs—a GRA25-BirA* fusion and a previously unpublished GRA13 (TgME49_237880)-BirA* fusion (see Fig. 2 below for IFA of GRA13-HA). These constructs were expressed in *Toxoplasma* (see Fig. S1 in the supplemental material), and then the *in vivo* biotinylation and purification were performed as in the GRA17-BirA* experiment. We again found by tandem mass spectrometry (MS/MS) analysis that many of the known components of the PV were purified and scored high by spectral count and unique peptide count (Table 2 and Table 3; see Tables S2 and S3 in the supplemental material). When we examined our collective data sets for common proteins, we found substantial (but not complete) overlap between these data sets (see Table S4 in the supplemental material). Between the three GRA-BioID experiments, we identified most of the known numbered GRAs and named dense granule proteins, including TgPSD1, NTPase II, PI-I, cyclophilin, several MAF1 proteins, and MYR1 (Tables 1, 2, and 3; see Tables S1 to S4). We were not able to detect GRAs 10, 19, 20, and 21 nor the recently described TgLCAT (22).

**TABLE 2:** Summary of known and novel GRAs found by GRA25-BioID^a^

Gene ID (TgGT1 no. [ToxoDB version 7.3])	Gene ID (TgGT1 no. [ToxoDB release 25])	Protein name
108770.0	270240	MAG1
032340	290700	GRA25
017550	203310	GRA7
083030	227620	GRA2
004030	254470	MYR1
**017570**	**203290**	**GRA34**
102940.0	251540	GRA9
021860	208830	GRA16
**081550**	**226380**	**GRA35**
051100	239740	GRA14
068065	275440	GRA6
**115760**	**232000**	**GRA30**
068050	275470	GRA15
087090	310780	GRA4
**039660**	**213067**	**GRA36**
096230	297880	GRA23
108780	270250	GRA1
**014920**	**220240**	**GRA31**
**069040**	**236890**	**GRA37**
082670	227280	GRA3
000480	275860	GRA12
004270	254720	GRA8
053890	410360	MAF1-related protein
125960	215220	GRA22
053780	410370, 279100, 279100A, 279100B, 411470, 220950	MAF1-related protein
**033840**	**289380**	**GRA39**
**115800**	**231960**	**GRA28**
065990	222170	GRA17
**108120**	**269690**	**GRA29**
117710	230180	GRA24
**088710**	**312420**	**GRA38**

a Novel GRAs 28-40 are highlighted in boldface.

**TABLE 3:** Summary of known and novel GRAs found by GRA13-BioID^a^

Gene ID (TgGT1 no. [ToxoDB version 7.3])	Gene ID (TgGT1 no. [ToxoDB release 25])	Protein name
083030	227620	GRA2
058380	237880	GRA13
017550	203310	GRA7
108770.0	270240	MAG1
034740	288650	GRA12
108780.0	270250	GRA1
**017570**	**203290**	**GRA34**
004030	254470	MYR1
087090	310780	GRA4
125960.0	215220	GRA22
**072150**	**212300**	**GRA32**
051100	239740	GRA14
096230	297880	GRA18
021470	208450	TgPI-1
032340	290700	GRA25
102940	251540	GRA9
**088710**	**312420**	**GRA38**
**069040**	**236890**	**GRA37**
**033840**	**289380**	**GRA39**
**014920**	**220240**	**GRA31**
004270	254720	GRA8
**115760**	**232000**	**GRA30**
021860	208830	GRA16
**030810**	**219810**	**GRA40**
082670	227280	GRA3
**039660**	**213067**	**GRA36**
064850	410370, 279100, 279100A, 279100B, 411470, 220950	MAF1-related protein
053890	410360	MAF1-related protein
**115800**	**231960**	**GRA28**
**108120**	**269690**	**GRA29**
**081550**	**226380**	**GRA35**
068050	275470	GRA15

a Novel GRAs 28-40 are highlighted in boldface.

We also analyzed the data from the GRA-BioID data sets for potential interacting or proximal host proteins. In each of the experiments, a relatively small number of host proteins were identified (21 for GRA17, 43 for GRA25, and 93 for GRA13). This could be due to the relative abundance of parasite versus host targets within the vacuole or the fact that few proteins are actually in close contact with the BirA* fusions. Only a single host protein, programmed cell death 6-interacting protein, was in common between the all three GRA-BioID experiments. Other common human hits between pairs of BioID experiments are shown in Table S5 in the supplemental material.

### Identification of novel dense granule proteins from BioID data sets.

Analysis of the GRA-BioID data sets also yielded a large number of hypothetical proteins (see Tables S1, S2, and S3 in the supplemental material), which were filtered for likely GRAs by selecting proteins that contained a predicted signal peptide, were constitutively expressed, and lacked a C-terminal endoplasmic reticulum (ER) retention signal (K/HDEL) (7, 8). As most GRAs identified to date also lack similarity to known proteins, we also selected candidates that lacked obvious functional domains for verification. This resulted in a list of >100 candidates, of which we chose 15 for localization studies using endogenous gene tagging (see Tables S1, S2, and S3).

To assess localization of these candidate proteins, we engineered constructs that would recombine a sequence encoding a 3×HA tag at the C terminus of each gene’s product and localized the HA signal by IFA. Of the 15 proteins tested, 13 trafficked to the PV and colocalized with the dense granule protein GRA14 (TgGT1_231960, TgGT1_269690, TgGT1_232000, TgGT1_220240, TgGT1_212300, TgGT1_247440, TgGT1_203290, TgGT1_226380, TgGT1_213067, TgGT1_236890, TgGT1_312420, TgGT1_289380, and TgGT1_219810) (Fig. 2). We therefore named these proteins GRAs 28 to GRA40, respectively (Tables 1, 2, and 3; see Tables S1 to S4 in the supplemental material). Intriguingly, GRA28 (TgGT1_231960) was exported to the host cell and trafficked to the host nucleus (Fig. 2B). *In silico* analysis revealed that this protein indeed contained a predicted nuclear localization sequence at residues 400 to 410. This protein now joins the new class of exported dense granule proteins that traffic to the host nucleus, along with GRAs 16 and 24. Examination of these proteins by BLAST analysis revealed that GRA38, GRA39, and GRA40 shared sequence similarity, although GRA38 and GRA39 were closer to one another (data not shown). This analysis also detected two other proteins not found in the GRA-BioID data sets with similarity to GRAs 38 to 40 (TgGT1_293500 and TgGT1_250790). As TgGT1_293500 contained a predicted signal peptide, we tagged this protein, which showed that TgGT1_293500 appeared to localize the endoplasmic reticulum (data not shown). While TgGT1_250790 was closest in similarity to GRAs 38 and 39, it did not contain a signal peptide and so was not further studied. BLAST analysis also revealed low levels of similarity between GRAs 35 and 36. Through our verification process, we also tagged 2 other proteins (of 15) from the GRA-BioID data sets which did not localize to the PV: TgGT1_209720 localized to the mitochondrion, while TgGT1_304990 localized to the Golgi apparatus (see Table S1) (data not shown). The remaining candidate proteins that match our criteria for potential GRAs have yet to be localized. However, the high frequency of successful GRA proteins in our verification analysis suggests that many of the remaining candidates are likely to be novel GRAs.

### GRAs 38, 39, and 40 are not tightly associated with PV membranes.

**FIG 3:**
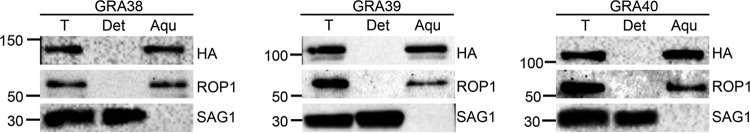
Triton X-114 fractionation of GRAs 38, 39, and 40. Triton X-114 (T) phase partitioning shows that GRA38, GRA39, and GRA40 are preferentially found in the aqueous (Aqu [soluble]) phase with the soluble marker ROP1, whereas the glycosylphosphatidylinositol (GPI)-anchored SAG1 remained in the detergent (Det [membrane]) fraction. Detergent lysis and fractionation were performed on intracellular parasites.

We chose to analyze the group of GRAs 38, 39, and 40, as GRA38 was previously found to be antigenic in a human serological screen that was performed in collaboration with our lab (23). Most of the previously described GRA proteins are associated with the parasitophorous vacuolar membrane or the intravacuolar network in *Toxoplasma* infections. GRAs 38, 39, and 40 lack predicted transmembrane domains or other readily identifiable sequences for membrane association, suggesting they are soluble components of the vacuole. To assess this experimentally, we performed Triton X-114 partitioning of intracellular parasites and found that each of these family members fractionated in the aqueous fraction (as opposed to the detergent/membrane fraction), demonstrating that they are not firmly associated with the IVN or PV membrane (PVM) (Fig. 3). While we cannot exclude the possibility of weak associations with vacuolar membranes, these data indicate that this new family of GRA proteins are soluble proteins of the PV.

### Targeted disruption of GRAs 38, 39, and 40.

**FIG 4:**
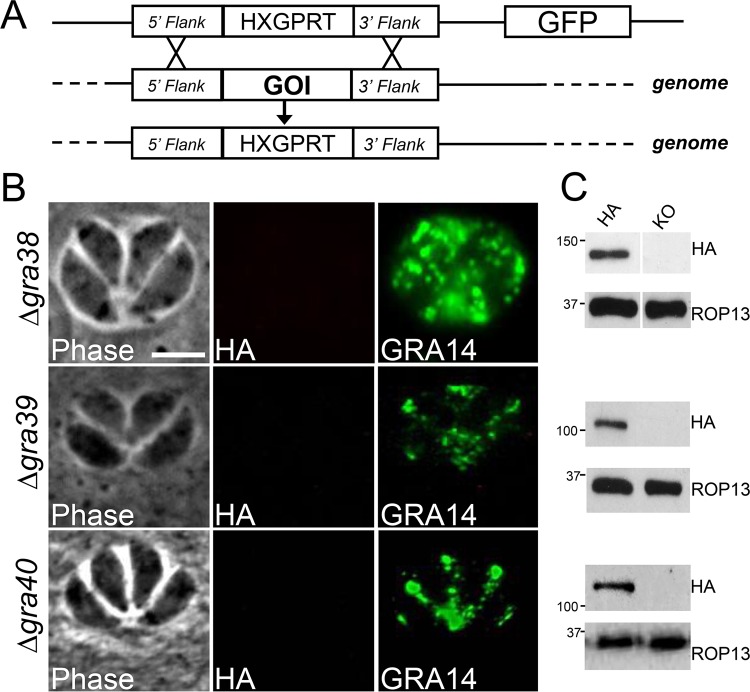
Targeted disruption of the gene encoding GRAs 38, 39, and 40. (A) Schematic depicting the GRA knockout strategy. Using homologous recombination, the GRA coding region was replaced with the selectable marker gene *HXGPRT*. (B) IFA of Δ*gra38*, Δ*gra39*, and Δ*gra40* parasites demonstrating a lack of HA staining, but not GRA14, in the PV. (C) Western blot analysis of lysates of Δ*gra38*, Δ*gra39*, and Δ*gra40* parasites and their respective parental strains. ROP13 is used as a loading control. KO, knockout.

**FIG 5:**
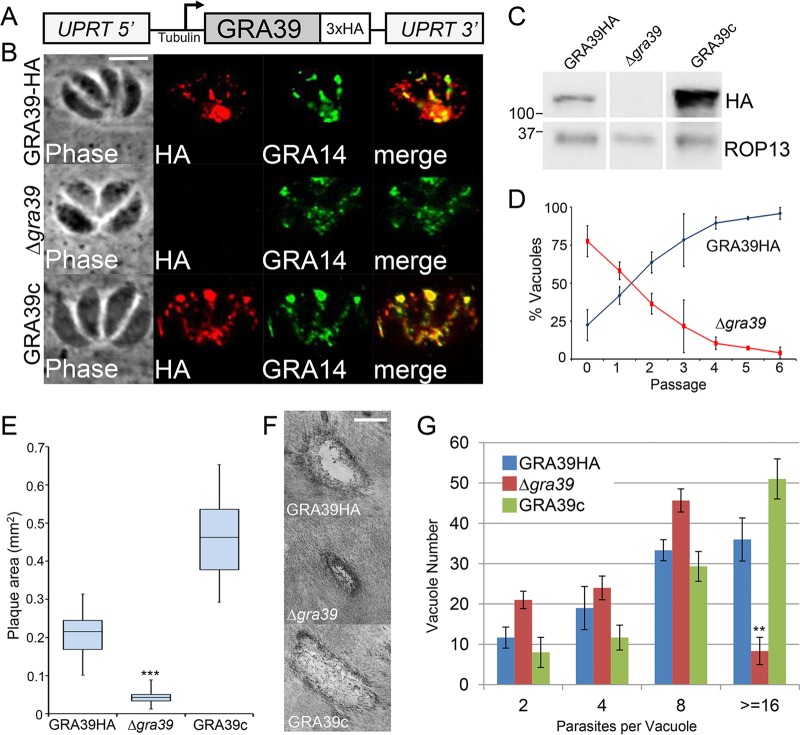
Disruption of *GRA39* results in a slow-growth phenotype of *Toxoplasma in vitro*. (A) Complementation strategy of *GRA39* into the *UPRT* locus. The complemented gene contains a C-terminal 3×HA epitope tag and is driven by the tubulin promoter. (B) IFA of GRA39-HA, Δ*gra39*, and GRA39c parasites. Rabbit anti-HA shows strong staining in the vacuole of GRA39-HA and GRA39c parasites, colocalizing with GRA14. HA signal is absent in Δ*gra39* parasites. Scale bar, 10 µm. (C) Western blot demonstrating GRA39 migrating as a single band at the predicted size of ~103 kDa (size of GRA39 after signal peptide cleavage and the addition of the 3×HA tag). GRA39c parasites demonstrate a significant overexpression of the protein driven by the tubulin promoter. (D) Competition assay of GRA39-HA and Δ*gra39* parasites. Parasites were initially mixed in an 80/20 ratio (HA/knockout) and then passaged serially after lysis of the host cell monolayer. Vacuoles from the mixed population were enumerated at each passage. Each data point represents 100 random vacuoles counted, with 3 replicates per data point. Error bars indicate 3× the standard deviation. (E) Quantitation of plaques formed by GRA39-HA, Δ*gra39*, and GRA39c parasites. Results are shown as box-whisker plots, with the middle line representing the median, the bottom and top of the box representing the 25th and 75th percentiles, respectively, and the whiskers corresponding to smallest and largest plaques. Each bar of the graph represents an average of means across experimental replicates (*n* = 3 for all conditions), and two-sample two-tailed *t* tests were performed comparing each condition to GRA39-HA to assess statistical significance (***, *P* = 0.0006). (F) Phase microscopy images of representative plaques made by GRA39-HA, Δ*gra39*, and GRA39c parasites in HFF monolayers. Cells were fixed in methanol and stained with crystal violet. Scale bar, 500 µm. (G) Counts of parasites/vacuole done after 36 h of infection of GRA39-HA, Δ*gra39*, and GRA39c parasites. A total of 100 vacuoles were quantitated per parasite line, with three replicate counts completed. Bars represent mean parasite/vacuole count, with error bars showing 1 standard deviation from each mean. Statistical significance comparing each line to GRA39-HA was calculated for ≥16 parasites/vacuole using a two-sample two-tailed *t* test (**, *P* = 0.003). The occurrence of 1 parasite/vacuole was not counted for any line, as it could not be determined whether or not these were viable events.

To understand the function of the newly characterized GRAs, we disrupted *GRA38*, *GRA39*, and *GRA40* by homologous recombination in their respective 3×HA-tagged parental strains (Fig. 4A). Knockout clones were identified that lacked the 3×HA tag by IFA (Fig. 4B) and Western blotting (Fig. 4C) and were verified by PCR (see Fig. S2 in the supplemental material). Intriguingly, Δ*gra39* clones were initially difficult to isolate from a transfected population as they were quickly outcompeted by nonhomologous recombinants, suggesting these parasites possessed a growth phenotype *in vitro*. However, we were able to obtain a knockout clone that we subsequently complemented with a construct containing HA-tagged GRA39 driven from the tubulin promoter (Fig. 5A). This transgene was targeted to the uracil phosphoribosyltransferase locus (*UPRT*) (24), and its expression was confirmed by IFA and Western blotting using both anti-HA antibodies and anti-GRA39 polyclonal antibodies. (The complemented strain was designated GRA39c [Fig. 5B and C; see Fig. S3 in the supplemental material].)

### GRA39 is critical for efficient growth *in vitro* in type II *T. gondii*.

Next, we analyzed the overall fitness of our GRA knockout parasites using competition growth assays and plaque assays. Competition growth assays showed that Δ*gra39* parasites were rapidly outcompeted by their GRA39-HA parental line after 7 serial passages (Fig. 5D). Plaque assays similarly revealed that Δ*gra39* mutants produced significantly smaller plaques (~80% reduction in size) than the GRA39-HA line in human foreskin fibroblast (HFF) monolayers (Fig. 5E and F). This poor-growth phenotype in knockout parasites was reversed upon complementation of *GRA39*. To investigate this phenotype further, we examined the rate of division of parasites during intravacuolar growth. We found that the Δ*gra39* line replicated at a lower rate than GRA39-HA and GRA39c parasites and also had a substantial number of parasites that appeared to have died early upon invasion (see below). At the end of 36 h, we found that ~8% of Δ*gra39* parasite vacuoles contained ≥16 parasites per vacuole, compared to 36% for GRA39-HA vacuoles (Fig. 4G). Consistent with our plaque assay results, this reduced replication rate was reversed upon complementation. Of the Δ*gra39* vacuoles that were replicating, we observed no apparent organellar defects (e.g., dense granule, apicoplast, mitochondria, rhoptries, or IMC) or dysregulation of endodyogeny as examined by IFA at various time points (data not shown). Together, these data indicate that loss of *GRA39* severely disrupts parasite growth *in vitro*. Similarly, Δg*ra38* and Δ*gra40* parasites were analyzed by competition and plaque assays, but these strains showed no apparent growth or replication defects (not shown).

### Deletion of GRA39 results accumulation of lipids in the PV, generation of amylopectin granules in the parasite cytoplasm, and parasite death.

**FIG 6:**
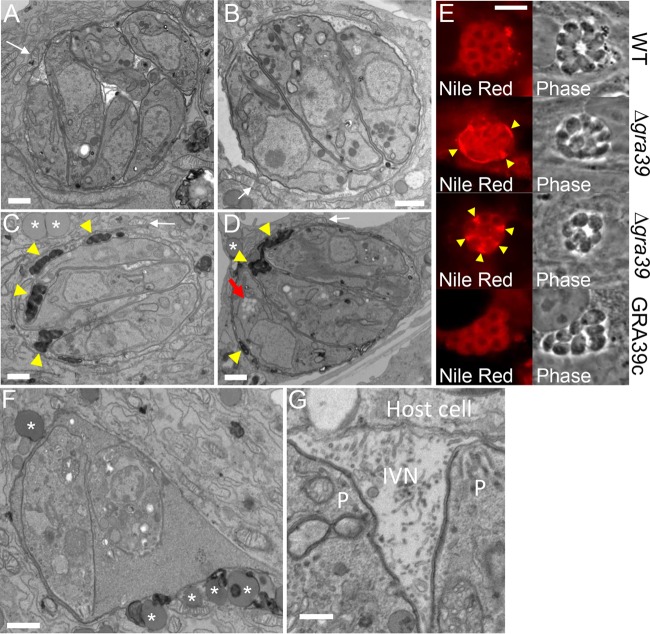
Deletion of GRA39 results in accumulation of lipid deposits in the PV, generation of amylopectin granules in the parasite cytosol. (A to D) Transmission electron micrographs demonstrating wild-type (A), GRA39c (B), and Δ*gra39* (C and D) parasites within parasitophorous vacuoles. The Δ*gra39* line shows accumulation of osmiophilic lipid deposits within the parasitophorous vacuole (yellow arrowheads) and amylopectin granules within the parasite cytosol (red arrow in panel D). Increased amounts of lipid droplets were also found in the host cytosol proximal to the PV (asterisks). The association of mitochondria with the PV remained intact in all three lines (white arrows). Scale bars, 500 nm. (E) Nile Red staining of intracellular parasites, once again demonstrating an accumulation of neutral lipids within the PV (Δ*gra39*, yellow arrowheads). (F) Transmission electron micrograph of Δ*gra39* parasites showing signs of death in an enlarged PV. (Host lipid vacuoles are indicated by asterisks.) Scale bar, 500 nm. (G) Transmission electron micrograph of intracellular Δ*gra39* parasites illustrating the normal ultrastructure of the IVN, close to the PVM, between two parasites (P). Scale bar, 250 nm.

To better examine the phenotype of the GRA39 knockout on an ultrastructural level, we examined intracellular wild-type, Δ*gra39*, and GRA39c parasites by transmission electron microscopy (Fig. 6). We found that Δ*gra39* parasites accumulated dramatic osmiophilic lipid deposits within the parasitophorous vacuole (Fig. 6C and D, yellow arrowheads). We were also able to visualize lipid material in the PV by fluorescence staining with the lipophilic dye Nile Red (Fig. 6E). These lipid deposits were not present in either wild-type or GRA39c parasites (Fig. 6A, B, and E). We additionally noticed that some of the Δ*gra39* parasites had increased levels of amylopectin granules in the cytoplasm of the parasite (Fig. 6D, red arrow) and also observed the clustering of host lipid vacuoles around the PV (Fig. 6C, D, and F, asterisks). The GRA39 knockout also resulted in a substantial number of vacuoles that contained dying parasites, as evidenced by loss of plasma membrane integrity, poorly identifiable intracellular structures, and abnormal enlargement of the PV space (Fig. 6F). These dying parasites could often be observed alongside healthy parasites with normal PVs in the same host cell, indicating that the host microenvironment was not the cause of parasite death (not shown). In knockout parasites that appeared to replicate within vacuoles (albeit more slowly), organelles, including the dense granules, exhibited normal morphology. The ultrastructure of the intravacuolar network was intact (Fig. 6G), and the presence of host organelles such as mitochondria associated with the PVM was also unaffected by *GRA39* deletion (Fig. 6A to D, white arrows).

As GRA38 is closest in sequence similarity to GRA39 and may play a similar role in *Toxoplasma* infections, we attempted to generate a GRA38 GRA39 double knockout. To do this, we first removed the *HXGPRT* gene from the Δ*gra38* parasite by homologous recombination (see Fig. S3 in the supplemental material) and then attempted to delete *GRA39* from the Δ*gra38* Δ*hxgprt* strain. We attempted to generate the double knockout using both homologous recombination and clustered regularly interspaced short palindromic repeats (CRISPR) with Cas9, screening populations and individual clones using our polyclonal rat anti-GRA39 antibody. After multiple attempts with different strategies (see Materials and Methods), we were unable to isolate a viable *GRA38 GRA39* deletion mutant, suggesting that the tandem loss of these genes is lethal to the parasite.

### GRA39 is critical for virulence in mice.

**FIG 7:**
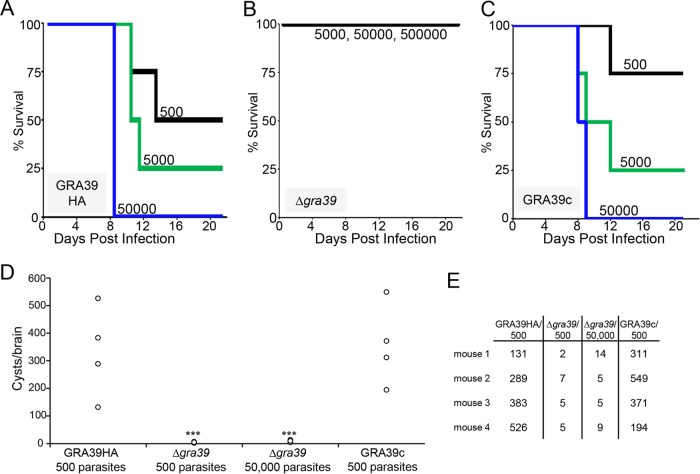
GRA39 is critical for virulence and normal brain cyst burden in mice. (A to C) Groups of 4 C57BL/6 female mice each were infected with indicated doses of GRA39-HA (A), Δ*gra39* (B), and GRA39c (C) parasites, and survival was monitored. Kaplan-Meier survival curves were generated. Mice infected with GRA39-HA and GRA39c parasites died in a dose-dependent manner over time (A and C). However, all mice infected with Δ*gra39* parasites survived throughout the full 21 days of the observation period (B). Surviving mice were euthanized after 30 days of observation. Mantel-Cox log rank test was used for the following comparisons: GRA39-HA parasites at 5,000 parasites/mouse versus Δ*gra39* parasites at 5,000, 50,000, or 500,000 parasites/mouse, *P* = 0.039; GRA39-HA at 50,000 parasites/mouse versus Δ*gra39* at 50,000 or 500,000 parasites/mouse, *P* = 0.008. (D and E) Bradyzoite cysts were quantitated from brains extracted from CBA/J mice sacrificed after 30 days of infection with GRA39-HA, Δ*gra39*, and GRA39c parasites. (Groups of 4 mice per experiment were used.) Although Δ*gra39* parasites were able to establish a chronic infection, the cyst burden was greatly decreased compared to that in infection with the GRA39-HA and GRA39c lines. Statistical significance of the difference in cyst burden between strains and infections was calculated using a two-sample two-tailed *t* test comparing all experiments to GRA39-HA (500 parasites/mouse) infection: ***, *P* = 0.03.

To evaluate whether the growth defect of Δ*gra39* mutants observed *in vitro* translated into a virulence defect *in vivo*, we carried out parallel infections of GRA39-HA, Δ*gra39*, and GRA39c parasites in C57BL/6 mice. Infected mice were observed for symptoms of infection, weight loss, and morbidity. Mortality for mice infected with the GRA39-HA parental line was consistent with previously published virulence studies with type II parasites, with a 50% lethal dose (LD_50_) of ~10^3^ to 10^4^ (25) (Fig. 7A). Mice infected with a dose of 50,000 GRA39-HA parasites exhibited 100% mortality by day 11 postinfection. In sharp contrast, mice infected with either 5,000, 50,000, or 500,000 Δ*gra39* parasites all survived to the end of the experiment (21 days) (Fig. 7B). Symptoms of infection were also less severe in mice infected with Δ*gra39* parasites. These mice generally moved with less impairment, had less ruffled coats, and lost less weight than mice infected with the parental and GRA39c lines (see Fig. S4 in the supplemental material). As with *in vitro* experiments, this virulence defect reverted upon complementation of GRA39 (Fig. 7C).

To evaluate the role of GRA39 during the chronic infection, we examined brain cyst burden in surviving mice after 30 days. While the parental Pru Δ*ku80* Δ*hxgprt* strain makes low numbers of cysts in C56BL/6 mice, higher cyst numbers can be obtained in CBA/J mice (26, 27). Thus, we repeated the infections in CBA/J mice with GRA39-HA, Δ*gra39*, and GRA39c parasites and examined the brains from the euthanized mice after 30 days of infection. Using this approach, we were able to quantitate the number of bradyzoite cysts and found that brains from Δ*gra39* strain-infected mice contained approximately 100-fold-lower numbers of cysts than those infected with GRA39-HA and GRA39c lines, even with a 100-fold-higher inoculum of the Δ*gra39* strain (Fig. 7D and E). The cysts obtained from Δ*gra39* parasites appeared to be morphologically normal upon gross examination (not shown), suggesting that the defect is limited to the cyst burden in Δ*gra39* parasites, rather than viability of the cysts themselves.

We next examined the ability of this line to form bradyzoite cysts *in vitro* using pH shift (pH 8.1) (28). We found that Δ*gra39* parasites were able to form *in vitro* cysts as efficiently as GRA39-HA and GRA39c parasites (data not shown). In addition, we noticed that the GRA39 signal at the PV was more faint by IFA in *in vitro* encysted GRA39-HA bradyzoites *in vitro* than in tachyzoites, in agreement with a downregulation of *GRA39* transcription during the bradyzoite stage (29, 30), suggesting that this protein likely does not have an important role in chronic infection. Together, these data demonstrate that GRA39 likely serves a role in the normal replication of parasites and that the Δ*gra39* mutant has difficulty establishing a chronic infection due to its inability to replicate at a normal rate.

## DISCUSSION

The parasitophorous vacuole serves as an interface between the parasite and its host cell (8, 17). The vacuolar membrane is semipermeable due to a membrane pore comprised of GRAs 17 and 23, which allows for the exchange of small molecules (~1,300 to 1,900 Da), enabling uptake of nutrients from the host cell (10). GRAs 17 and 23 are also related to the *Plasmodium* translocon component EXP2, and thus they may also play roles in protein export from the PV into the host cell (10). Inside the PV, GRAs 2, 4, and 6 participate in the formation of the IVN, which is believed to traffic nutrients as well as maintain a semirigid supporting scaffold for the vacuole (9, 12, 13). An array of other GRAs are present in the PV, PVM, and IVN, but their precise roles in intracellular survival are not well defined. As many of these GRAs are not conserved outside *Toxoplasma* and its closest relatives (e.g., *Neospora* and *Hammondia*), their functions are likely specialized for the intracellular niche of this group, rather than common functions of apicomplexan parasites as a whole (6). Uncovering the proteome of the PV can therefore not only reveal the survival mechanisms of *T. gondii* but also may expose a number of novel parasite-specific drug targets.

In spite of its central role in *Toxoplasma* growth and survival, the PV has been challenging to study due to excessive parasite and/or host contamination using traditional biochemical fractionation approaches (31, 32). The BioID method has enabled us to specifically label the PV constituents by *in vivo* biotinylation, therefore circumventing the need for fractionations (19, 20). We initially chose GRA17 as the bait protein because it is highly expressed, and thus we envisioned that it would be able to label a broad array of proteins in the PV. GRA17 is proposed to function in conjunction with its paralog GRA23 at the PVM, as either hetero- or homomultimeric complexes (10). In agreement with this, GRA23 scored highly in the mass spectrometry data set (Table 1; see Table S1 in the supplemental material). However, a number of other GRAs ranked highly in our data set, thus differentiating between interacting and proximal proteins will require independent experiments. We also examined the GRA17-BioID hits for other proteins that might be similar to the *Plasmodium* PTEX complex (e.g., heat shock protein 101 [HSP101] and thioredoxin 2) (33–36). While the HSP-related ER protein BIP (HSP70 [TgGT1_311720]) (37, 38) was labeled in our experiment, no other putative HSPs containing signal peptides were found that might represent a vacuolar heat shock protein similar to HSP101. It is likely that BIP was labeled by GRA17-BirA*, while the fusion protein trafficked through the secretory pathway, as evidenced by the biotinylation of proteins such as signal peptidase (TgGT1_300060). We did identify four thioredoxin-like proteins containing predicted signal peptides (TgGT1_209950, TgGT1_204480, TgGT1_247350, and TgGT1_224060), none of which have been characterized. In addition, we identified GRA16 and GRA24, both of which are exported into the host cell and transit to the host nucleus, as well as GRA15 and MAF1, which are inserted into the vacuolar membrane and are exposed to the host cytoplasm (15, 16, 39, 40). Like GRA23, the extent to which these are truly proteins that interact with GRA17 or merely proximal at the vacuolar membrane remains to be determined.

To evaluate the extent of vacuolar labeling with different GRA bait proteins, we replicated the GRA17-BioID experiment with GRA25 and GRA13. These GRA-BioID experiments also labeled a wide array of previously known and novel GRA proteins. As expected, the overlap between these experiments was not perfect, with some GRAs appearing only in one experiment or another (e.g., GRA5 was found in the GRA17-BirA* experiment alone, and GRAs 17, 12, 24 appeared only in GRA17- and GRA25-BirA* experiments). While the data suggest broad labeling of the vacuole, the ranking of individual proteins (by NSAF) in the data sets likely reflects both their proximity to the GRA-BirA* fusion proteins and their overall abundance. The high success rate of our verifications by endogenous gene tagging supports specific labeling of GRAs secreted into the PV. Many of the novel GRAs contain predicted transmembrane domains (GRAs 30, 31, 33, 35, and 36). This is expected, as many GRAs anchor into the IVN or PV membrane, and both the GRA17 and GRA25 bait proteins are also membrane associated (8). Of these, GRAs 33 and 36 demonstrated a particularly strong IFA signal on the PV membrane (Fig. 2). Many GRAs are known to be antigenic as antibodies against these secreted proteins are found to be peptide ligands for major histocompatibility complex (MHC) class I receptors (41). Indeed, GRA4 and GRA7 have also been heavily researched as vaccine candidates. A recent examination of *Toxoplasma* peptide ligands to HLA-A*2:01 that 15% of verified ligands were GRAs, including MAG1 and NTPase II. Not surprisingly, we found in examining their data that novel GRAs 28, 29, 32, and 39 were also MHC class I ligands, further stressing the importance of these secreted proteins in the host immune response (41).

As expected, most of the GRAs identified were expressed predominantly in tachyzoites or equally in tachyzoites and bradyzoites. However, GRAs 30 and 34 are upregulated in bradyzoites, which may suggest that they play roles in the transition to bradyzoites or that their function is primarily in the cyst form of the parasite. Similarly, we found that the predominantly bradyzoite protein MAG1 (42) ranked very highly in our spectral analysis, suggesting that this protein is also present in tachyzoites. This finding agrees with *in vivo* studies and mass spectrometry data indicating MAG1 is expressed in the tachyzoite stage of the parasite (43). The use of bradyzoite GRA proteins as bait in encysted parasites in future BioID studies promises to be a powerful approach to identify secreted vacuolar proteins that function in the formation or maintenance of the bradyzoite cyst.

While most of the GRAs identified to date lack paralogs in *Toxoplasma* (8), we identified a group of new GRA proteins (GRAs 38 to 40) that have shared sequence similarity. The levels of similarity are relatively low, with GRAs 38 and 39 being the most similar to one another (E value, 6 × 10^−10^), whereas GRA40 is more distantly related to both GRAs 38 and 39 (E values, 2 × 10^−4^ and 7 × 10^−4^, respectively). As is observed with many GRAs, these proteins lack identifiable domains that might provide clues toward their function, and they are unique to *Toxoplasma* and its closest coccidian relatives (e.g., *Hammondia*, *Neospora*, and *Eimeria*). It is noteworthy that GRAs 38 to 40 are significantly larger than most previously identified GRAs (~100 kDa versus the 30 to 50 kDa typical of most GRA proteins) and appear to be soluble components of the PV (Fig. 3) (8). We addressed the function of these new players by gene disruption and showed that none of these proteins is individually essential for parasite survival. However, we were unable to generate a double knockout of GRAs 38 and 39, indicating at least partial functional redundancy and these two most closely related players are essential for parasite survival.

Disruption of GRA39 resulted in a strong growth defect and the appearance of striking deposits of lipid material in the PV, which suggests that GRA39 could function in the utilization of lipids from the vacuolar space into the parasite, although other effects on vacuolar lipid regulation are also possible. The accumulation of host lipid droplets around the PV may be additional evidence linking the phenotype of the *GRA39* knockout with parasite lipid metabolism. The Δ*gra39* parasites also showed an increase in amylopectin granules, which are present during times of nutritional stress and in which the parasite relies on gluconeogenesis to acquire carbon (44, 45). These granules are often seen in the slow-growing bradyzoite stage of the parasite and thus may be linked to slow growth in the Δ*gra39* parasites (although it is important to note that the knockout does not show enhanced switching to bradyzoites). If GRA39 is indeed important for metabolism, this would also explain our observation that a small percentage of knockout parasites die shortly after invasion; this would likely be due to starvation as the parasite is unable to acquire the nutrient it needs to complete endodyogeny. The dramatic reduction in virulence in the knockout is likely a consequence of the slow growth of the mutant strain, as well as a higher rate of parasite death, which together enable the host to better control the infection. Disruption of GRA39 also has an effect on the chronic infection, which displays a sharply reduced brain cyst burden during infections *in vivo*. This may be due to changes in tissue tropism in the knockout or more simply another effect of the growth defect in the knockout.

The development of GRA39 knockout parasites also provides a foundation for identification of domains important for function by mutagenesis and subsequent complementation, which will help to determine the precise role of this protein in *Toxoplasma* growth and virulence. Together, the adaptation of the BioID approach for the identification of novel GRAs and the discovery of GRAs 38 to 40 open new insight into the arsenal of GRAs that enable *Toxoplasma* to survive in its intracellular niche and cause disease.

## MATERIALS AND METHODS

### Host cell and parasite cultures.

*T. gondii* strains RH Δ*hxgpt* (type I, strain RH), Pru Δ*ku80* Δ*hxgprt* (type II, strain Prugniaud), and modified strains were used to infect human foreskin fibroblast (HFF) cells. HFFs were grown in Dulbecco’s modified Eagle’s medium supplemented with 10% fetal bovine serum and 2 mM glutamine and maintained as previously described (46).

### Antibodies.

The primary antibodies used in immunofluorescence assays (IFAs) and Western blotting were mouse polyclonal anti-GRA14 (1:1,000) (21), rabbit anti-HA (Covance [1:2,000]), rabbit polyclonal anti-ROP13 (1:500) (47), and rat polyclonal anti-GRA39 (1:1,000). For generation of anti-GRA39 antisera, the full coding region of *GRA39* was PCR amplified from Prugniaud strain cDNA using primers p41 and p42 (see Table S6 in the supplemental material). This product was ligated in frame into pet-28a, which contains both N-terminal and C-terminal polyhistidine tags, using EcoRI and NotI and transformed into BL21 Star(DE3) chemically competent *Escherichia coli* cells. Protein expression was induced with IPTG (isopropyl-β-d-thiogalactopyranoside) and purified using Ni-nitrilotriacetic acid (NTA) agarose beads under denaturing conditions. The eluted protein was then dialyzed to remove urea and injected into a rat for antibody production (Cocalico Biologicals).

### IFA and Western blotting.

For IFA, HFFs were grown to confluence on coverslips and infected with *T. gondii* parasites. After 18 to 36 h, the coverslips were fixed and processed for indirect immunofluorescence as previously described (32). Primary antibodies were detected by species-specific secondary antibodies conjugated to Alexa Fluor 594/488. The coverslips were mounted in ProLong Gold antifade reagent (Thermo, Fisher) and viewed with an Axio Imager Z1 fluorescence microscope (Zeiss) as previously described (21). For Western blotting, parasites were lysed in Laemmli sample buffer (50 mM Tris-HCl [pH 6.8], 10% glycerol, 2% SDS, 1% 2-mercaptoethanol, 0.1% bromophenol blue), and lysates were resolved by SDS-PAGE and transferred onto nitrocellulose membranes. Blots were probed with the indicated primary antibodies, followed by secondary antibodies conjugated to horseradish peroxidase (HRP). Target proteins were visualized by chemiluminescence.

### Nile Red staining.

Confluent HFF monolayers on coverslips were infected with *T. gondii* parasites. Thirty-six hours postinfection, the coverslips were fixed in 4% paraformaldehyde and stained with 0.5 µg/ml Nile Red as previously described (48, 49). The coverslips were then mounted onto slides in Vectashield antifade reagent and viewed as described above.

### Transmission electron microscopy.

HFFs infected with the wild-type Prugniaud, Δ*gra39*, or GRA39c strain for 26 h were fixed in 2.5% glutaraldehyde in 0.1 M sodium cacodylate buffer (pH 7.4) for 1 h at room temperature and processed as described (50) before examination with a Philips CM120 electron microscope (Eindhoven, The Netherlands) under 80 kV.

### Generation of GRA17-, GRA25-, and GRA13-BirA* fusions.

To generate the GRA17-BirA* fusion, the full *GRA17* gene and promoter were PCR amplified from genomic DNA (primers p1 and p2; see Table S6 in the supplemental material). This was cloned into the p.LIC.BirA*.3×HA.DHFR vector by ligation independent cloning as previously described (20). Thirty micrograms of the construct was linearized with HindIII and transfected into RH Δ*hxgprt* parasites. The parasites were selected with medium containing 1 µM pyrimethamine, cloned by limiting dilution, and screened by IFA and Western blotting against the HA tag. A clone expressing the fusion protein was selected and designated GRA17-BirA*. To generate the GRA25-BirA* and GRA13-BirA* fusions, the procedure described above was replicated using the PCR-amplified genomic regions of the respective genes. For GRA25-BirA*, ~900 bp of the 3′ genomic region was used (p5 and p6); for GRA13-BirA* the whole of the genomic region was used along with a portion of the promoter (p3 and p4). GRA25-BirA* and GRA13-BirA* constructs were transfected into Pru Δ*ku80* Δ*hxgprt* parasites, and clones were generated as described above.

### Affinity capture of biotinylated proteins.

HFF monolayers were infected with parasites expressing BirA* fusions or the respective parental line (RH Δ*hxgprt* for GRA17-BirA* and Pru Δ*ku80* Δ*hxgprt* for GRA13- and GRA25-BirA*) for 12 h and then grown in medium containing 150 µM biotin for an additional 24 h (20). Infected cells were collected by manual scraping, washed in phosphate-buffered saline (PBS), and lysed in radioimmunoprecipitation assay (RIPA) buffer (50 mM Tris [pH 7.5], 150 mM NaCl, 0.1% SDS, 0.5% sodium deoxycholate, 1% NP-40) supplemented with Complete protease inhibitor cocktail (Roche) for 30 min on ice. Lysates were centrifuged for 15 min at 14,000 × *g* to pellet insoluble material, and the supernatant was incubated with Streptavidin Plus UltraLink resin (Pierce) at room temperature for 4 h under gentle agitation. Beads were collected and washed five times in RIPA buffer, followed by three washes in 8 M urea buffer (50 mM Tris-HCl [pH 7.4], 150 mM NaCl). Ten percent of each sample was boiled in Laemmli sample buffer, and eluted proteins were analyzed by Western blotting by streptavidin-HRP, while the remainder was used for mass spectrometry.

### Mass spectrometry of biotinylated proteins.

Purified proteins bound to streptavidin beads were reduced, alkylated, and digested by sequential addition of trypsin protease (51, 52). The peptide mixture was desalted using C_18_ tips and fractionated online using a 75-µm-inner-diameter fritted fused silica capillary column with a 5-µm pulled electrospray tip and packed in house with 15 cm of Luna C_18_ (2) 3-µm reversed-phase particles. The gradient was delivered by an easy-nLC 1000 ultrahigh-pressure liquid chromatography (UHPLC) system (Thermo Scientific) (53, 54). Tandem mass spectrometry (MS/MS) spectra were collected on a Q-Exactive mass spectrometer (Thermo Scientific). Data analysis was performed using the ProLuCID and DTASelect2 implemented in the Integrated proteomics pipeline IP2 (Integrated Proteomics Applications, Inc., San Diego, CA) (55, 56). Protein and peptide identifications were filtered using DTASelect and required a minimum of two unique peptides per protein and a peptide-level false-positive rate of less than 5%, as estimated by a decoy database strategy (57). Normalized spectral abundance factor (NSAF) values were calculated as described previously (58).

### Generation of GRA-HA parasites (epitope tagging of BioID hits).

Candidate GRA proteins identified by mass spectrometry were selected based on the presence of a signal peptide using SignalP 4.1 software (http://www.cbs.dtu.dk/services/SignalP/). Candidates were further narrowed for those with constitutive cell cycle RNA expression profiles in tachyzoites and lacked C-terminal endoplasmic reticulum localization sequences (K/HDEL or similar). To epitope tag the novel GRAs, ~1,500 bp of the 3′ genomic region of each gene was amplified using the primers listed in Table S6 in the supplemental material (primers p7-p28, p33-p34, and p43-44) from Pru Δ*ku80* Δ*hxgprt* genomic DNA. The products were cloned into the p.LIC.3×HA.DHFR vector to add a 3×HA tag on the target gene as previously described (59–61). The constructs were linearized, and 50 µg of DNA was transfected by electroporation into the Pru Δ*ku80* Δ*hxgprt* strain of *T. gondii*. The transfected parasites were grown in medium containing 1 µM pyrimethamine, and selected parasites were cloned by limiting dilution. Clones were designated “GRA(X)-HA,” where “X” represents the protein number (e.g., GRA30-HA). All confirmed GRAs were examined *in silico* by BLAST to search for other *Toxoplasma* proteins with sequence similarity.

### Triton X-114 detergent extraction.

Intracellular parasites were lysed in precondensed Triton X-114. Precondensation was performed as previously described (62). Briefly, after 20 min of lysis on ice, the insoluble portion was removed by high-speed centrifugation (3,000 × *g* for 10 min at 4°C). Subsequently, the lysate was warmed to 30°C for 5 min to achieve the cloud point. Micelles (detergent fraction) were then separated from the aqueous fraction by centrifugation of the lysate at 30°C (3,000 × *g* for 10 min). The fractions were examined by SDS-PAGE and Western blotting for the HA-tagged target proteins, using SAG1 and ROP1 as the detergent and aqueous controls, respectively.

### Deletion of the genes encoding GRA38, GRA39, and GRA40.

For disruption of GRAs 38, 39, and 40, genomic sequences flanking the respective genes were amplified from *T. gondii* Pru Δ*ku80* Δ*hxgprt* strain genomic DNA and subcloned into the p.mini-GFP.hxgprt.ht vector (for the sequences of primers p29-32, p35-39, and p45-50, see Table S6 in the supplemental material) (59, 60). This vector contains the selectable marker hypoxanthine-xanthine-guanine phosphoribosyl transferase gene (*HXGPRT*) and a green fluorescent protein (GFP) cassette located downstream for negative selection of heterologous recombinants. The final constructs were linearized, and 50 µg of DNA was transfected by electroporation into the respective parental HA-tagged strain. The transfected parasites were grown in medium containing 50 µg/ml MPA (mycophenolic acid) and 50 µg/ml xanthine and cloned by limiting dilution. GFP-negative parasites were then screened by IFA and confirmed by Western blotting using mouse anti-HA antibodies. The knockouts were further verified by PCR (p27-28, p39, p43-44, and p49-55 in Table S6 and see Fig. S3 in the supplemental material). To attempt to generate a Δ*gra38* Δ*gra39* mutant, we first removed the *HXGPRT* cassette from the p.mini.gra38 knockout plasmid (see Fig. S3A). We then transfected this plasmid into Δ*gra38* parasites, and selected *HXGPRT*-negative clones using 6-thioxanthine selection. Loss of *HXPGPRT* was confirmed by PCR (p49 and p50 in Table S6 and see Fig. S2A in the supplemental material). Subsequently, we attempted to delete GRA39 in the Δ*gra38* Δ*hxgprt* mutant using three separate strategies. We first attempted to isolate a clone by transfecting the p.mini.gra39.hxgprt construct used for the single knockout. Second, we attempted to use CRISPR-Cas9 using a guide RNA (gRNA) directed toward the *GRA39* locus and introducing an *HXGPRT* cassette to replace the entire coding region using homologous recombination with 5′ and 3′ flanks of *GRA39* as previously described (63). Finally, we again attempted CRISPR-Cas9 deletion, again using a gRNA directed toward the first intron of *GRA39*; however, this time replacing a portion of the coding sequence with 3 in-frame stop codons and introducing a frameshift mutation using an 80-bp double-stranded oligonucleotide (64).

### Complementation of GRA39.

GRA39-HA was reintroduced into the genome of the Δ*gra39* line in the uracil phosphoribosyltransferase locus (*UPRT*) (24). This was done by amplifying the GRA39-3×HA gene from genomic DNA of the GRA39-HA parasite line. The PCR product was cloned into the *UPRT* knockout vector with a tubulin promoter added using PacI and NsiI, forming a construct with the GRA39-3×HA cassette inserted between the *UPRT* locus 5′ and 3′ flanking regions (see Table S6, primers p39-40). This plasmid was linearized using XbaI and transfected into Δ*gra39* parasites and selected with 5-fluoro-5′-deoxyuridine (FUDR) to isolate parasites with the construct integrated at the *UPRT* locus (65). Clones were selected by limiting dilution and examined by IFA (anti-HA antibody), and a positive clone was designated GRA38c.

### Growth competition/mixing assay.

GRA39-HA and Δ*gra39* parasites were mixed in an initial 20/80 ratio and passaged. After each lysis (an average of 48 to 60 h), the mixed population was passed into a new HFF monolayers and screened by IFA to examine the ratio of GRA39-HA to Δ*gra39* vacuoles. Three replicates of 200 vacuoles each were counted, and the percentage of HA versus knockout parasites was calculated (66).

### Plaque assay.

Serial dilutions of GRA39-HA, Δ*gra39*, and GRA39c parasites were used to infect separate wells of a 24-well plate with an HFF monolayer and allowed to form plaques. The dilutions were calibrated to infect at 20 parasites/well, 60 parasites/well, and 120 parasites/well. Six days after infection, HFF monolayers were fixed in 3.7% formaldehyde for 15 min, washed with PBS, dried, and stained with crystal violet. Plaques were then visualized with a Zeiss upright light microscope (Zeiss Axio Imager Z1). Plaque areas were measured using ZEN software (Zeiss), and GRA39-HA, Δ*gra39*, and GRA39c plaque sizes were compared. Three separate experiments with 30 plaques for each parasite line were measured to generate a mean plaque size along with interquartile ranges. Statistical significance comparing the GRA39-HA line to the other strains was calculated using a two-sample two-tailed *t* test.

### Parasite-per-vacuole assay.

HFF coverslips were infected separately with GRA39-HA, Δ*gra39*, and GRA39c parasites, and incubated for 36 h. The samples were then fixed in 100% methanol and stained using primary mouse anti-SAG1 antibody and FITC-conjugated secondary goat anti-mouse secondary antibody. Vacuoles were examined for parasite count (2, 4, 8, or ≥16 parasites/vacuole) and scored. In total, 100 vacuoles were counted per parasite line. This experiment was repeated in triplicate to generate a mean and standard deviation (67), and statistical significance between the strains was calculated for ≥16 parasites/vacuole using a two-sample two-tailed *t* test.

### Mouse virulence assays.

Intracellular GRA39-HA, Δ*gra39*, and GRA39c parasites mechanically liberated from infected HFF monolayers and resuspended in Opti-MEM reduced serum medium (Thermo, Fisher Scientific) prior to intraperitoneal injection into groups of 4 female C57BL/6 mice per line per dose (e.g., 4 mice were injected each with 500 GRA39-HA parasites, 4 mice each injected with 5,000 GRA39-HA parasites, etc.). Injected parasites were confirmed to be live and viable by plaque assays with HFF monolayers. Mice were monitored for symptoms of infection, weight loss, and mortality for 21 days. Survival was plotted on a Kaplan-Meier curve. Surviving mice were sacrificed at 30 days after infection, and mouse brains were collected in aseptic fashion, homogenized, and examined by phase microscopy and fluorescence microscopy (for detection of GFP under the *LDH2* promoter for parasite encystation). In parallel, 1/3 of each brain was homogenized and incubated with HFF monolayers to qualitatively assess viability of parasites.

### Mouse brain cyst quantitation.

Intracellular GRA39-HA, Δ*gra39*, and GRA39c parasites were mechanically liberated from infected HFF monolayers and resuspended in Opti-MEM prior to intraperitoneal injection into groups of 4 female CBA/J mice each. A total of 500 parasites/mouse of GRA39-HA and GRA39c and two separate doses (500 parasites/mouse and 50,000 parasites per mouse) of the Δ*gra39* strain were injected. The mice were monitored for 30 days after infection and then sacrificed. Mouse brains were collected, homogenized, and examined for *T. gondii* cysts (as described above). Quantitation of cysts was performed by examining 25-µl aliquots of homogenate by fluorescence microscopy until approximately 25% (by volume) of each brain was examined. Total cyst burden was then extrapolated. The statistical significance of cyst burden between GRA39-HA, Δ*gra39*, and GRA39c experiments was calculated by two-sample 2-tailed *t* test.

### Ethics Statement.

Antibodies were raised in rats under the guidelines of the Animal Welfare Act and the PHS Policy on Humane Care and Use of Laboratory Animals. Specific details of our protocol were approved by the UCLA Institutional Animal Care and Use Committee, known as the Chancellor's Animal Research Committee (protocol # 2004-055).

## SUPPLEMENTAL MATERIAL

Figure S1  GRA25- and GRA13-BirA* localize to the PV and can biotinylate proteins in the vacuole. IFA of GRA25- and GRA13-BirA*-expressing parasites ± biotin shows the PV is labeled in a biotin-dependent manner. Endogenously biotinylated apicoplasts are observed with and without biotin. Scale bar, 10 µm. Download Figure S1, TIF file, 2.7 MB

Figure S2  Gene deletion constructs and verification of gene deletions by PCR. Schematic for the deletions of *GRA38* (A), *GRA39* (B), and *GRA40* (C) using the p.mini vector and double-crossover homologous recombination, along with PCR products shown confirming the absence of the target genes and the presence of the deletion constructs. (Primer numbers refer to the primers found on Table S6 in the supplemental material). The *GRA38* locus was first deleted using p.mini.hxgprt, followed by a deletion of the *HXGPRT* cassette (A). Download Figure S2, TIF file, 2.9 MB

Figure S3  Polyclonal rat anti-GRA39 labels the novel dense granule protein GRA39. (A) IFA showing that rat anti-GRA39 staining of the PV overlaps with anti-GRA7 staining in Pru Δ*ku80* Δ*hxgprt* parasites. (B) Western blot showing rat anti-289380 detects a single band at ~97 kDa, which is the predicted size of GRA39 after removal of the 35-amino-acid N-terminal signal sequence. Download Figure S3, TIF file, 2.9 MB

Figure S4  GRA39 mouse infection weights and survival. Graphs show mouse weights (grams) for C57BL/6 mice injected intraperitoneally with the designated doses of GRA39-HA, Δ*gra39*, and GRA39c parasites. Each mouse is designated by a separate color line on a given chart. A weight of “0 g” indicates a mouse that was euthanized due to extreme illness. Download Figure S4, TIF file, 2.9 MB

Table S1  List of GRA17-BioID hits found by mass spectrometry. Known vacuolar components are shown in blue. Newly discovered GRAs found in this study are shown in red. Sheet 1 shows the experimental data set listed in rank order by NSAF score (excluding control hits, but including those hits shared by the experimental and control whose spectral count were greater than 2-fold higher under the experimental condition). Sheet 2 shows the full MS/MS data set: experimental, control, and shared set, along with NSAF × 10^5^ scores and peptide counts. Table S1, XLSX file, 0.1 MB

Table S2  List of GRA25-BioID hits found by mass spectrometry. Known vacuolar components are shown in blue. Newly discovered GRAs found in this study are shown in red. Sheet 1 shows the experimental data set listed in rank order by NSAF score (excluding control hits, but including those hits shared by the experimental and control whose spectral count were greater than 2-fold higher under the experimental condition). Sheet 2 shows the full MS/MS data set: experimental, control, and shared set, along with NSAF × 10^5^ scores and peptide counts. Table S2, XLSX file, 0.1 MB

Table S3  List of GRA13-BioID hits found by mass spectrometry. Known vacuolar components are shown in blue. Newly discovered GRAs found in this study are shown in red. Sheet 1 shows the experimental data set listed in rank order by NSAF score (excluding control hits, but including those hits shared by the experimental and control whose spectral count were greater than 2-fold higher under the experimental condition). Sheet 2 shows the full MS/MS data set: experimental, control, and shared set, along with NSAF × 10^5^ scores and peptide counts. Table S3, XLSX file, 0.1 MB

Table S4  Common proteins found by GRA17-, GRA13-, and GRA25-BioID MS/MS analysis. Common hits between all three experiments (experimental data sets, excluding proteins found in control lysates), as well as common hits found between pairs of BioID experiments are shown on separate sheets. Table S4, XLSX file, 0.1 MB

Table S5  Human protein hits from GRA17, GRA13, and GRA25-BioID mass spectrometry. The human host proteins identified by mass spectrometry (UniProt designation) with each of the BioID bait proteins are shown. Sheet 1, GRA17-BioID; sheet 2: GRA25-BioID, sheet 3, GRA13-BioID, sheet 4, common peptide hits between the BioID data sets (comparing the experimental data alone, excluding controls). Table S5, XLSX file, 0.1 MB

Table S6  Primers used in this study. All primers designated with “LIC” were used for generation of C-terminal endogenous tagging constructs using ligation-independent cloning as previously described (59–61). Primers used for PCR and standard ligation using restriction endonucleases are shown with the endonuclease site underlined. For all constructs stably transfected into *Toxoplasma*, the restriction endonuclease used to linearize the construct is listed. Table S6, DOCX file, 0.03 MB
