# Supplementary figures and images for: In Vivo Biotinylation of the Toxoplasma Parasitophorous Vacuole Reveals Novel Dense Granule Proteins Important for Parasite Growth and Pathogenesis
Source: mBio. 2016 Aug 2;7(4):e00808-16. doi: 10.1128/mBio.00808-16 (PMC4981711; doi:10.1128/mBio.00808-16)

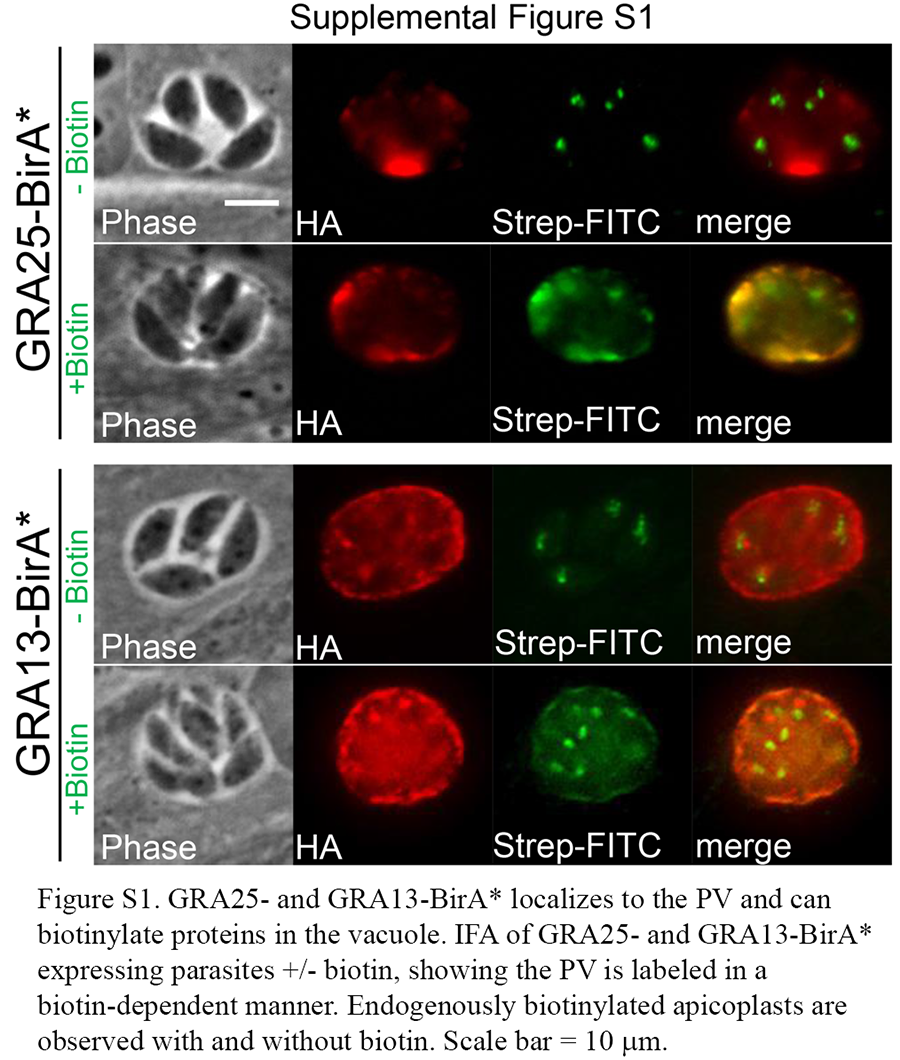

Supplement: Figure S1 — GRA25- and GRA13-BirA* localize to the PV and can biotinylate proteins in the vacuole. IFA of GRA25- and GRA13-BirA*-expressing parasites ± biotin shows the PV is labeled in a biotin-dependent manner. Endogenously biotinylated apicoplasts are observed with and without biotin. Scale bar, 10 µm. Download [file mbo004162909sf1.tif]

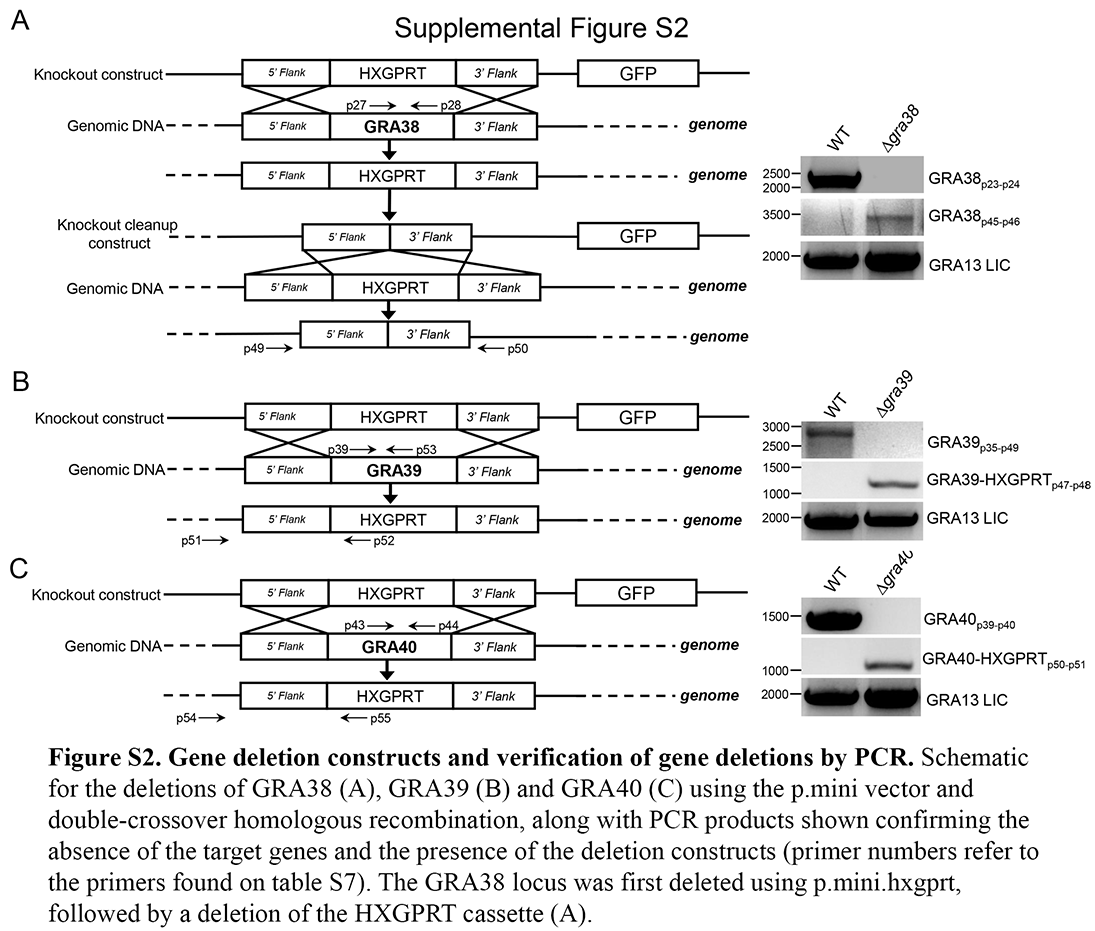

Supplement: Figure S2 — Gene deletion constructs and verification of gene deletions by PCR. Schematic for the deletions of GRA38 (A), GRA39 (B), and GRA40 (C) using the p.mini vector and double-crossover homologous recombination, along with PCR products shown confirming the absence of the target genes and the presence of the deletion constructs. (Primer numbers refer to the primers found on Table S6 in the supplemental material). The GRA38 locus was first deleted using p.mini.hxgprt, followed by a deletion of the HXGPRT cassette (A). Download [file mbo004162909sf2.tif]

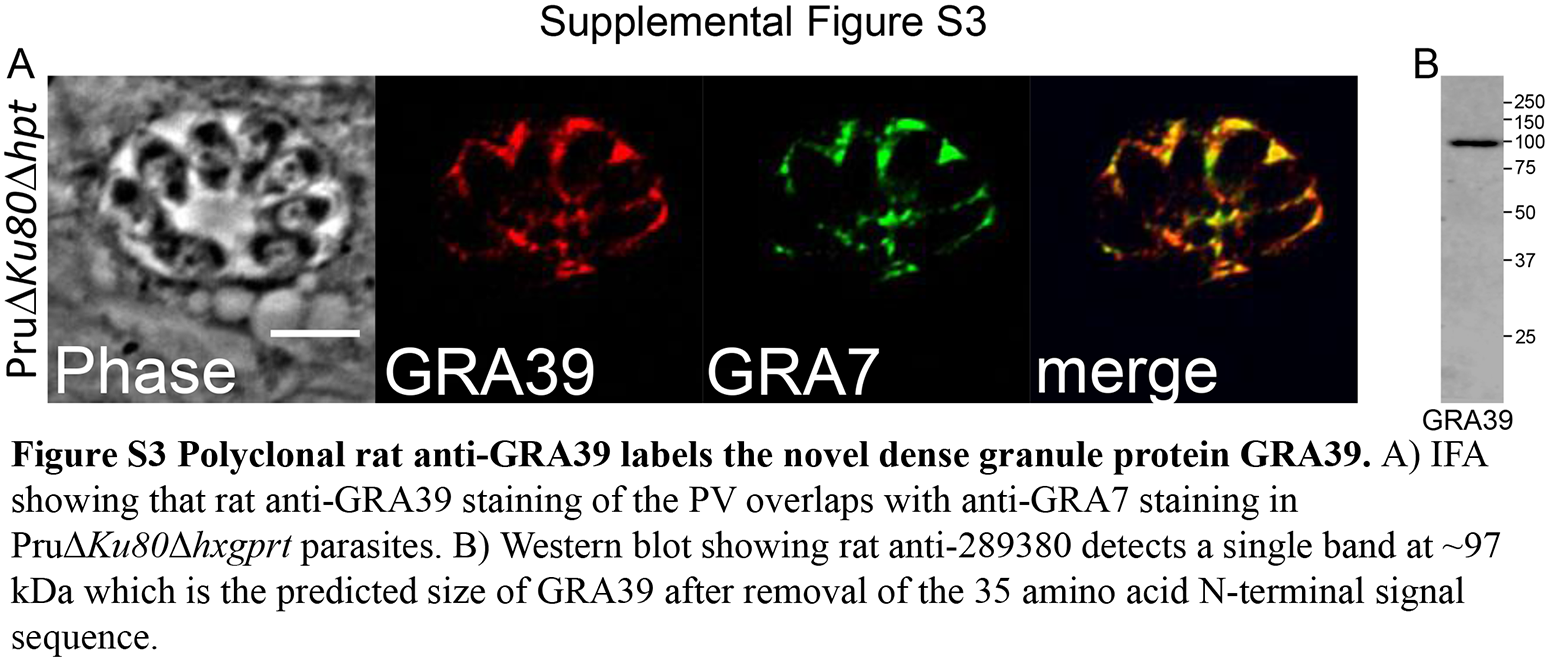

Supplement: Figure S3 — Polyclonal rat anti-GRA39 labels the novel dense granule protein GRA39. (A) IFA showing that rat anti-GRA39 staining of the PV overlaps with anti-GRA7 staining in Pru Δku80 Δhxgprt parasites. (B) Western blot showing rat anti-289380 detects a single band at ~97 kDa, which is the predicted size of GRA39 after removal of the 35-amino-acid N-terminal signal sequence. Download [file mbo004162909sf3.tif]

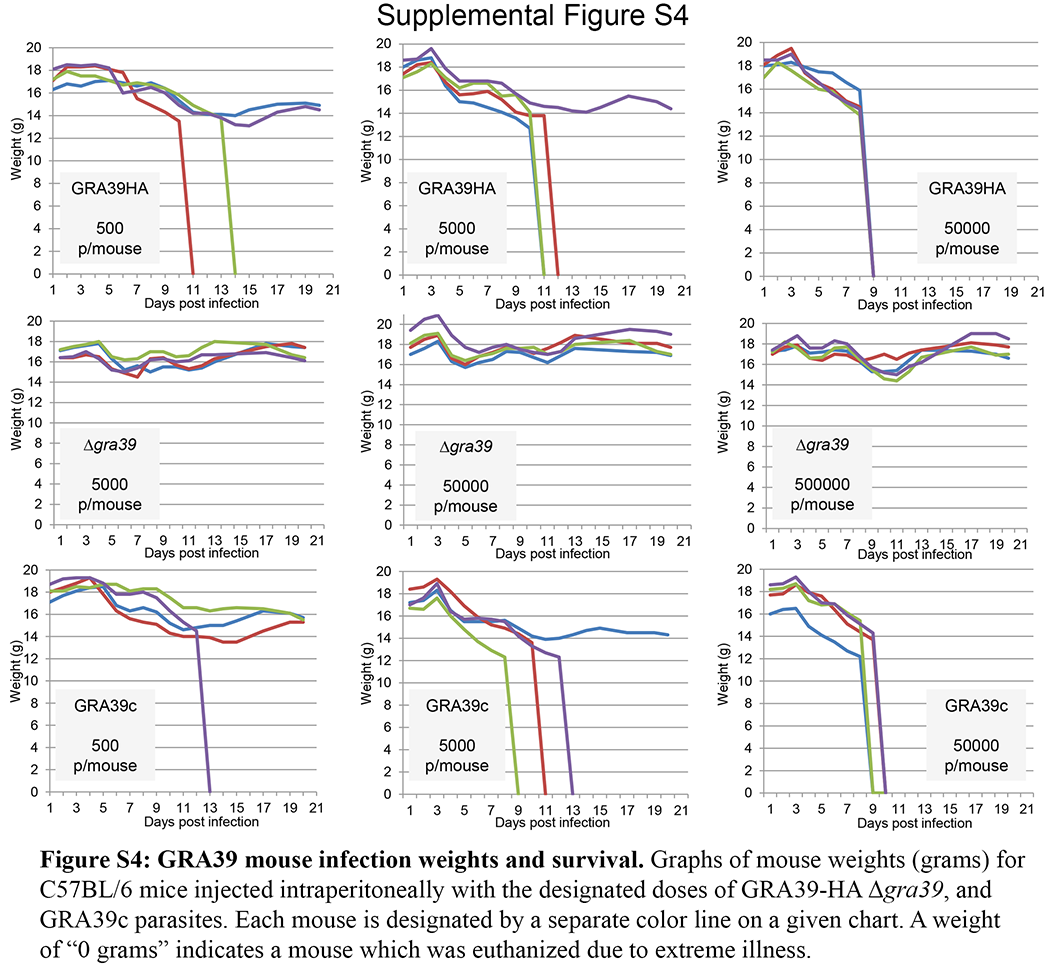

Supplement: Figure S4 — GRA39 mouse infection weights and survival. Graphs show mouse weights (grams) for C57BL/6 mice injected intraperitoneally with the designated doses of GRA39-HA, Δgra39, and GRA39c parasites. Each mouse is designated by a separate color line on a given chart. A weight of “0 g” indicates a mouse that was euthanized due to extreme illness. Download [file mbo004162909sf4.tif]
