# Supplementary material for: In Vivo Biotinylation of the Toxoplasma Parasitophorous Vacuole Reveals Novel Dense Granule Proteins Important for Parasite Growth and Pathogenesis
Source: mBio. 2016 Aug 2;7(4):e00808-16. doi: 10.1128/mBio.00808-16 (PMC4981711; doi:10.1128/mBio.00808-16)
Supplement: Table S6 — Primers used in this study. All primers designated with “LIC” were used for generation of C-terminal endogenous tagging constructs using ligation-independent cloning as previously described (59–61). Primers used for PCR and standard ligation using restriction endonucleases are shown with the endonuclease site underlined. For all constructs stably transfected into Toxoplasma, the restriction endonuclease used to linearize the construct is listed. [file mbo004162909st6.docx]

| **Supplemental Table S6**. Primers used in this study.  Name Description Sequence | | |  |
| --- | --- | --- | --- |
| p1 | GRA17 2nd copy LIC fwd | TAC TTC CAA TCC AAT TTA CGA TCG AAA TGT GTT TGG AAA TC |  |
| p2 | GRA17 2nd copy LIC rev | TCC TCC ACT TCC AAT TTT AGC CTG CTT GCC CTG CAT GGC |  |
| p3 | GRA13 LIC fwd | TAC TTC CAA TCC AAT TTA GTC GCA CTT CTG TTG TCG C |  |
| p4 | GRA13 LIC rev | TCC TCC ACT TCC AAT TTT AGC CTG GAA GTA TGT CGT GTC CG |  |
| p5 | GRA25 LIC fwd | TAC TTC CAA TCC AAT TTA GCG TTT CTG GTT GTG CGC |  |
| p6 | GRA25 LIC rev | TCC TCC ACT TCC AAT TTT AGC GTT TCT ATC GAA TTC CGG GAG |  |
| p7 | GRA28 (231960) LIC fwd | TAC TTC CAA TCC AAT TTA GCT CAC CAA GGA CAC ATC TG |  |
| p8 | GRA28 (231960) LIC rev | TCC TCC ACT TCC AAT TTT AGC TTC GGA ATA ACT GGA GCT ACC |  |
| p9 | GRA29 (269690) LIC fwd | TAC TTC CAA TCC AAT TTA GCC AAC AAG TTC TTC GAG AGC |  |
| p10 | GRA29 (269690) LIC rev | TCC TCC ACT TCC AAT TTT AGC ACG TGT CCC TCT TCC CAA C |  |
| p11 | GRA30 (232000) LIC fwd | TAC TTC CAA TCC AAT TTA GCC TGA AAG ACT ACA TCC TCG |  |
| p12 | GRA30 (232000) LIC rev | TCC TCC ACT TCC AAT TTT AGC GGT TCT AGT TCT TGC CGC ATC |  |
| p13 | GRA31 (220240) LIC fwd | TAC TTC CAA TCC AAT TTA CGT GTT AGC GCT CAC TTG C |  |
| p14 | GRA31 (220240) LIC rev | TCC TCC ACT TCC AAT TTT AGC CCT GCT GTC GTC TTG GTC T |  |
| p15 | GRA32 (212300) LIC fwd | TAC TTC CAA TCC AAT TTA GCG TAT ACT TGT TGC GTT TCG ACG |  |
| p16 | GRA32 (212300) LIC rev | TCC TCC ACT TCC AAT TTT AGC GGC CTC CGG GTG GCG AT |  |
| p17 | GRA33 (247440) LIC fwd | TAC TTC CAA TCC AAT TTA GCG GCA TCA AGA ACG GTT C |  |
| p18 | GRA33 (247440) LIC rev | TCC TCC ACT TCC AAT TTT AGC CGC CTT CGT GCT CTT CTT C |  |
| p19 | GRA34 (203290) LIC fwd | TAC TTC CAA TCC AAT TTA GCT ACG AGA CAC GCA GCG |  |
| p20 | GRA34 (203290) LIC rev | TCC TCC ACT TCC AAT TTT AGC CAC GGC AGA CGC GGC C |  |
| p21 | GRA35 (226380) LIC fwd | TAC TTC CAA TCC AAT TTA GCC GCC AAA TCA AAC TCC GA |  |
| p22 | GRA35 (226380) LIC rev | TCC TCC ACT TCC AAT TTT AGC AGT CTG TTT CGG CTC CGC C |  |
| p23 | GRA36 (213067) LIC fwd | TAC TTC CAA TCC AAT TTA GCA ACT GCG AAC AAA CCA GG |  |
| p24 | GRA36 (213067) LIC rev | TCC TCC ACT TCC AAT TTT AGC CGT ACG CTG TGC CCT TTC AA |  |
| p25 | GRA37 (236890) LIC fwd | TAC TTC CAA TCC AAT TTA GCT CTC AGC TGC AGT TGT G |  |
| p26 | GRA37 (236890) LIC rev | TCC TCC ACT TCC AAT TTT AGC TTC CAA GTC TGG GGG AAT ATC |  |
| p27 | GRA38 (312420) LIC fwd | TAC TTC CAA TCC AAT TTA GCA GCG CTA TCC AGT CGC C |  |
| p28 | GRA38 (312420) LIC rev | TCC TCC ACT TCC AAT TTT AGC CGC CTC GAA GGG GTT GCA C |  |
| p29 | GRA38 p.Mini 5' flank fwd | GCA TTC TAG AGC CAG TGA CTA TCG GTT CG |  |
| p30 | GRA38 p.Mini 5' flank rev | GCA TAC TAG TCG GTT TCT GCT GAG GCT TG |  |
| p31 | GRA38 p.Mini 3' flank fwd | GCT TCT CGA GCT CAC GCC TCT TGC AGG C |  |
| p32 | GRA38 p.Mini 3' flank rev | GTA CGG TAC CGT GGC ACT AGC TAT CGA GG |  |
| p33 | GRA39 (289380) LIC fwd | TAC TTC CAA TCC AAT TTA GCG CCC TTC ACG CGG TCG |  |
| p34 | GRA39 (289380) LIC rev | TCC TCC ACT TCC AAT TTT AGC CTG CGT CCC GCG GTT CAA CAA |  |
| p35 | GRA39 p.Mini 5' flank fwd | GCA TTC TAG AGC GGC CCA ACA AAC GAG C |  |
| p36 | GRA39 p.Mini 5' flank rev | CCG AAC TAG TCG ATC CTG GGC AGT GCT G |  |
| p37 | GRA39 p.Mini 3' flank fwd | GCA TCT CGA GCG TCC GAA AGT GGA CTG C |  |
| p38 | GRA39 p.Mini 3' flank rev | GCA TGG TAC CCC GGC AGT TAC AGA AAG CC |  |
| p39 | GRA39 Complement fwd | GCA TAG ATC TAT GGA GTC GAA TCG GCG AAG |  |
| p40 | GRA39 Complement 3XHA rev | GTA CTT AAT TAA TTA GGC ATA ATC TGG AAC ATC GTA |  |
| p41 | GRA39 pet28a FWD | GAA TTC CAG AAG AAC TCA GCC GAG C |  |
| p42 | GRA39 pet28a REV | GCG GCC GCG CGG TTC AAC AAG CCA CAG |  |
| p43 | GRA40 (219810) LIC fwd | TACTTCCAATCCAATTTAGGTCGAGACAGGAGAACGAG |  |
| p44 | GRA40 (219810) LIC rev | TCCTCCACTTCCAATTTTAGCCTTCTTTGAAGAATAGGAAACAAGATC |  |
| p45 | GRA40 p.Mini 5' flank fwd | GTA CGT TAA CGA CAC AGG CAC CGA ATA TGC |  |
| p46 | GRA40 p.Mini 5' flank rev | GCA TGC TAG CGC GAC AAG AAG TGA GCG CC |  |
| p47 | GRA40 p.Mini 3' flank fwd | GTC AGA ATT CCT GTG CAG CAT CAG TGG TC |  |
| p48 | GRA40 p.Mini 3' flank rev | GCA TGA TAT CCA GTG AGC CAC AGC AAG TG |  |
| p49 | GRA38 knockout check fwd | GCA GAC AGG AAC GAG TAG G |  |
| p50 | GRA38 knockout check rev | CGG CGT CTC TGT CTA CTC G |  |
| p51 | GRA39 knockout check 5’ fwd | CGC CAC GTT TAA CAT CCT AGC |  |
| p52 | p.mini knockout check HXGPRT rev | GAT AAT CTG CGA CCG CTG AAT C |  |
| p53 | GRA39 gene check rev | GCA TCC CGG GTG CGA AAG GTC G |  |
| p54 | GRA40 knockout check 5’ fwd | GGC TTC CTG CGA CTG GAA C |  |
| p55 | p.tKO knockout check HXGPRT rev | CGA ACA CGG TTA TCA AAC CCG |  |
